# Adnexal Torsion of a Mature Cystic Ovarian Teratoma With Hemorrhagic Infarction Misdiagnosed As Pelvic Inflammatory Disease in a Perimenopausal Patient: A Case Report

**DOI:** 10.7759/cureus.38680

**Published:** 2023-05-07

**Authors:** Efthymia Thanasa, Anna Thanasa, Emmanouil M Xydias, Apostolos C Ziogas, Evangelos Kamaretsos, Ioannis Paraoulakis, Vasiliki Grapsidi, Ektoras-Evangelos Gerokostas, Gerasimos Kontogeorgis, Ioannis Thanasas

**Affiliations:** 1 Department of Health Sciences, Medical School, Aristotle University of Thessaloniki, Thessaloniki, GRC; 2 Department of Obstetrics and Gynecology, EmbryoClinic IVF, Thessaloniki, GRC; 3 Department of Obstetrics and Gynecology, University of Thessaly, Larissa, GRC; 4 Department of Obstetrics and Gynecology, General Hospital of Trikala, Trikala, GRC

**Keywords:** mature cystic ovarian teratoma, dermoid cyst, adnexal torsion, imaging findings, adnexectomy, case report

## Abstract

Mature cystic teratomas are common benign ovarian tumors. They usually occur in young women, less than 40 years old. Our case report concerns a patient of perimenopausal age who came to the hospital complaining about mild abdominal pain, fever below 37.8°C, and diarrhea. The patient had an intrauterine contraceptive device inserted. Based on the clinical findings and imaging, a possible diagnosis of pelvic inflammatory disease was set, and intravenous administration of broad-spectrum antibiotics started immediately. The decision for performing laparotomy was taken after the fact that the clinical condition and blood tests of the patient had shown no improvement. Intraoperatively, the presence of a large twisted ovarian mass with signs of total necrosis due to adnexal torsion was detected. A histological examination of the surgical specimen confirmed the diagnosis of mature cystic teratoma in the right ovary. The postoperative course was uneventful. The presentation of the case follows a brief literature review of this rare medical condition regarding the diagnostic and therapeutic approach of these patients.

## Introduction

Teratomas are germ cell tumors. In their typical form, they consist mainly of well-differentiated tissues derived from the endoderm, mesoderm, and ectoderm. Teratomas can be mature cystic which are benign, or they can be immature and characterized by malignant potential [1]. The first report of mature cystic teratoma concerning the ovaries was made by Johannes Scultetus in 1659. Later in 1863, Rudolf Virchow introduced the term "teratoma," which comes from the Greek word "teras" which means monster/beast/huge/enormous. The most common localization is in the ovaries and testicles. On a few occasions, they can be located in the anterior mediastinum, sacrococcygeal region, or neck [2].

The mature cystic teratoma of the ovaries or dermoid cyst (synonym) is estimated to account for 10-20% of all ovarian neoplasms. Mature cystic ovarian teratomas in the majority of cases (more than 80%) occur in women of reproductive age, especially in women under 40 years of age [3]. These are usually benign tumors with a good prognosis. The rate of malignant transformation is estimated to be between 1% and 3% and usually corresponds to postmenopausal women [4]. In addition, in rare cases, mature cystic teratomas may rupture [5]. In contrast, adnexal torsion with mature cystic teratoma is common [6]. Adnexal torsion, especially when it is not expressed by signs of acute abdomen, as in our patient, can create a serious problem in the differential diagnosis and prevent early diagnosis. Ovarian torsion has a very variable clinical presentation. Pain can be dull or sharp, acute or not, intermittent or constant. Pain may be accompanied by nausea and vomiting, fever, and diarrhea less frequently.

With the occasion of the present case report, we must emphasize the difficulties that may arise in the differential diagnosis between torsion of mature cystic ovarian teratoma and pelvic inflammatory disease. Furthermore, we must underline the great importance of the early diagnosis and treatment of mature cystic ovarian teratomas complicated with complete adnexal torsion, especially when they concern young patients who wish to maintain the integrity of the female reproductive tract and achieve future pregnancy.

## Case presentation

**Figure 1:**
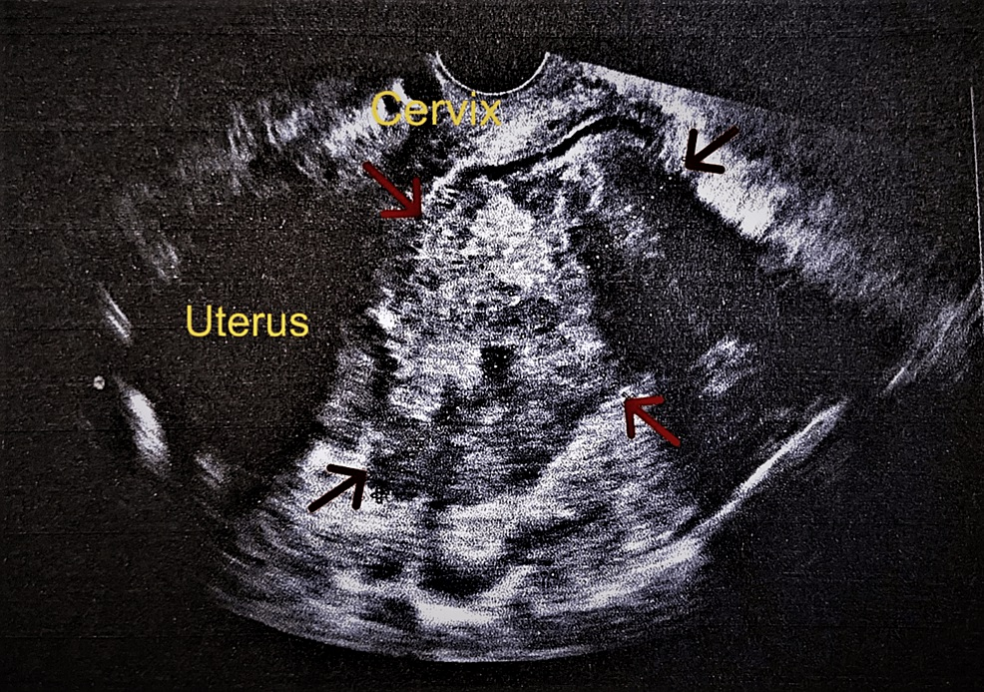
Transvaginal ultrasound imaging of a twisted mature cystic ovarian teratoma In the posterior pouch of Douglas, a solid inhomogeneous lesion is evident (red arrows), without cystic components and without the diagnostic imaging feature of intratumoral fat components forms that were misdiagnosed as a pelvic inflammatory disease.

A 49-year-old patient came to the emergency department of our hospital complaining about abdominal pain accompanied by a low fever (below 37.8°C). The onset of symptoms was five days ago. The location of the pain was in the lower abdomen, with mild intensity, without symptoms of peritoneal irritation. The patient did not report vomiting. She reported three bouts of diarrhea in the past two days. Her body temperature was 37.7°C, and her blood pressure and pulse rate were normal. The patient had three vaginal deliveries in her obstetric history, and her personal medical history was clear. She had an intrauterine contraceptive device placed four years ago and was removed during the gynecological examination and sent for culture. During the gynecological examination, a painful pelvic mass was found. Transvaginal ultrasound revealed a large inhomogeneous lesion of roughly 95x80 mm behind the uterus and in the pouch of Douglas, probably originating from the right adnexa (Figure 1). The left ovary was imaged without pathological findings.

**Figure 2:**
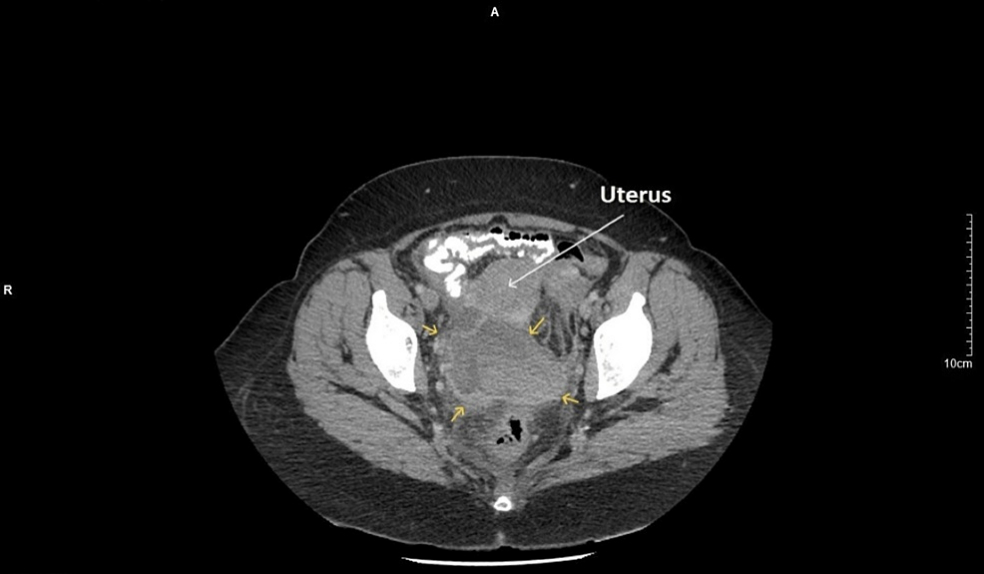
CT scan of a twisted mature cystic ovarian teratoma The posterior pouch of Douglas between the rectosigmoid and the uterus reveals the presence of a large mass (yellow arrows), which was misdiagnosed as an encapsulated pelvic inflammatory fluid collection that originated from the right adnexa.

The decision was made to admit the patient to our clinic and immediately start intravenous administration of broad-spectrum antibiotics to treat pelvic inflammatory disease. CT findings showed an enlarged edematous image of the right fallopian tube with opacification of the right ovary and a normal left ovary with the presence of small cystic follicles. More specifically, between the rectosigmoid and the uterus in the rectouterine pouch of Douglas, the presence of a large demarcated fluid collection with thick hyperintense walls and the presence of numerous septations in the pelvis around the fluid collection were depicted. The above imaging findings were probably attributed to an encapsulated pelvic inflammatory fluid collection that originated from the right adnexa (Figure 2).

The fact that non-improvement was made in the clinical condition and blood tests of our patient (Table 1) 48 hours later led to the decision to perform a laparotomy. Diagnostic laparoscopy was not available at our hospital.

**Table 1:** Laboratory tests of the patient on the day of entry to the clinic and after 48 hours of hospitalization Ht: hematocrit, Hb: hemoglobin, PLT: platelets, WBC: white blood cells, NEUT: neutral, CRP: C-reactive protein, APTT: activated partial thromboplastin time, INR: international normalized ratio, FIB: fibrinogen

Laboratory tests	Day of entry	48 hours later	Normal laboratory values
Ht	36.8%	33.1%	37.7-49.7%
Hb	12.1 gr/dl	10.9 gr/dl	11.8-17.8 gr/dl
PLT	287x10^3^/ml	264x10^3^/ml	150-350 x10^3^/ml
WBC	16.7x10^3^/ml	18.9x10^3^/ml	4-10.8 x10^3^/ml
NEUT	87.1%	93.4%	40-75%
CRP	13.7 mg/dl	21.5 mg/dl	<0.7 mg/dl
APTT	34.9 sec	39.2 sec	24.0-35.0 sec
INR	1.21	1.29	0.8-1.2
FIB	258 mg/dl	247 mg/dl	200-400 mg/dl

**Figure 3:**
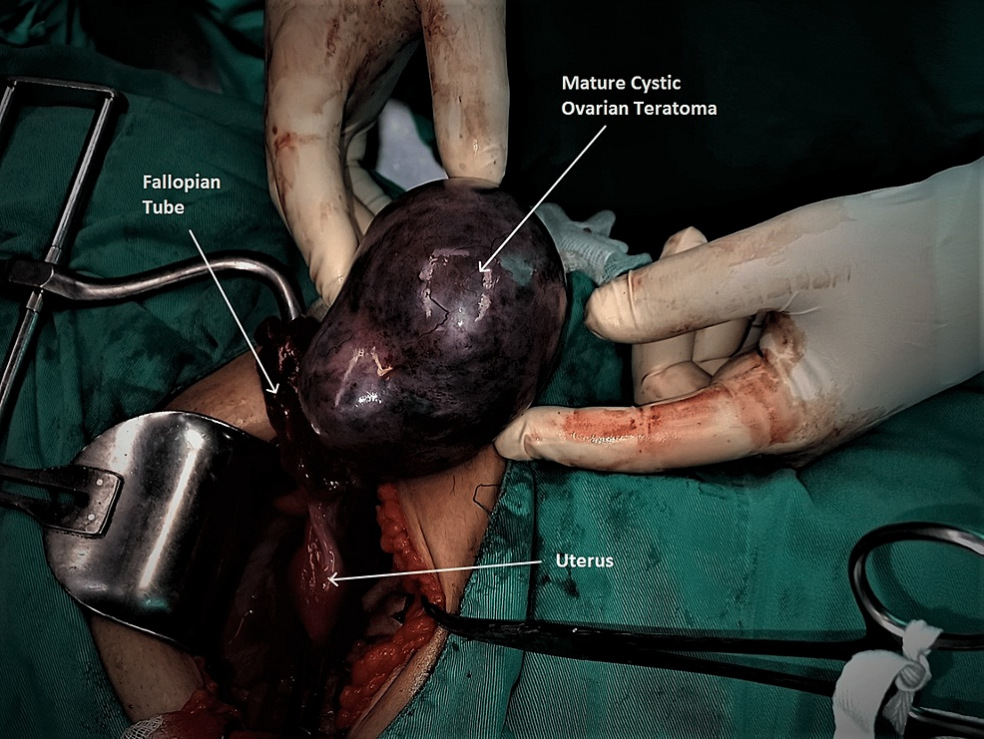
Intraoperative image of a twisted mature cystic teratoma of the ovary Adnexal torsion and total necrosis of the affected ovary and fallopian tube are evident.

Intraoperatively, the presence of a large twisted ovarian mass was found, originating from the right ovary and not adhering to the adjacent tissues. The mass had distinct signs of total necrosis due to ipsilateral adnexal torsion (Figure 3).

**Figure 4:**
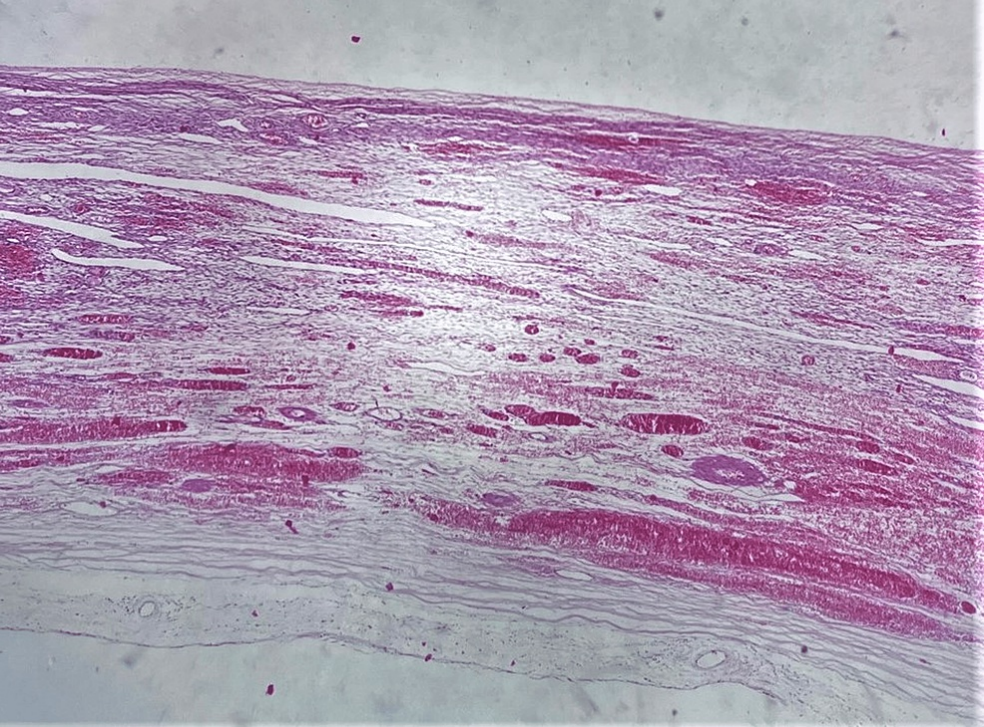
Histological image of a twisted mature cystic ovarian teratoma The presence of an ovarian dermoid cyst wall with hemorrhagic infiltration and edema as a result of torsion is depicted.

**Figure 5:**
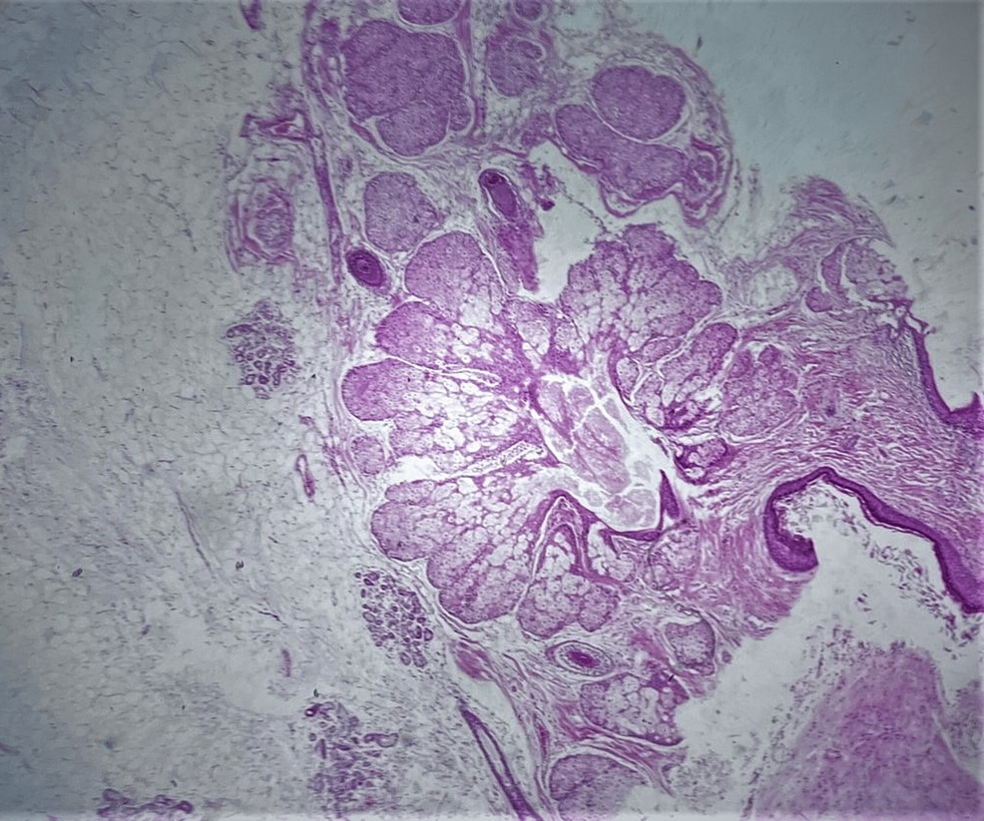
Histological image of a twisted mature cystic ovarian teratoma The wall of the dermoid cyst with skin, sebaceous glands, and fat tissue is depicted, without evidence of malignancy.

After detorsion of the twisted ovarian mass and the reveal of necrotic, non-viable tissues, adnexectomy was performed. Histological examination of the surgical specimen confirmed the diagnosis of mature cystic ovarian teratoma (Figures 4, 5).

On microscopic examination, a cyst with squamous epithelium, increased vascularity and edema, and sebaceous glands and hair follicles was observed. Adipose tissue and cerebral tissue were also observed. Immature tissue or malignancy was not observed. On the fourth postoperative day, the patient was discharged from the clinic with a consultation for re-examination at the gynecology outpatient clinic of our hospital.

## Discussion

Clinical diagnosis of mature cystic teratoma of the ovary is usually difficult. In most cases, mature cystic ovarian teratomas are asymptomatic. Although in ovarian torsion abdominal pain can have many variables, acute abdominal pain followed by vomiting may be associated with rupture of the ovarian mass or, more commonly, torsion of the ovarian pedicle [7]. In our case, the complete torsion of the ovarian pedicle was paradoxically not accompanied by sudden acute abdominal pain, nor by vomiting. The bouts of diarrhea, reported by our patient, were likely the result of bowel pressure from the pelvic inflammatory mass. The anatomical position of the mature cystic ovarian teratoma between the uterus and the rectosigmoid can explain the slow, incomplete twisting progression of the adnexa. Mild pain, a low fever, and increased inflammation were the results of this. Mild abdominal pain and low fever along with atypical imaging findings were misdiagnosed as pelvic inflammatory disease and development of tubal-ovarian abscess rather than complete adnexal torsion due to the presence of mature cystic ovarian teratoma. The misdiagnosis of pelvic inflammatory disease was further strengthened by the prolonged use of intrauterine contraceptive devices. As can be seen from the patient's history, the intrauterine contraceptive coil was maintained for more than one year beyond the recommended date. This significantly increases the risk of pelvic inflammatory disease. In addition, based on the clinical presentation and imaging findings, the increase in inflammatory markers was not associated with the adnexal torsion but was attributed to the development of pelvic inflammatory disease with tubal-ovarian abscess. As in cases of intra-abdominal inflammation-infection, similarly, in patients with adnexal torsion, the number of white blood cells, neutrophil percent, neutrophil count, and neutrophil-to-lymphocyte ratio are statistically significantly higher [8]. Furthermore, in 2017, Wang et al. showed that the combined measurement of CA125, CA19-9, and neutrophil-to-lymphocyte ratio significantly contributes to the diagnosis of twisted mature cystic ovarian teratomas [9].

The significance of imaging in the evaluation of ovarian masses and adnexal torsion remains determinant. On ultrasound, the mature cystic ovarian teratomas appear as a simple cystic mass without solid components up to a complex solid cystic mass with typical imaging features of intratumoral fat components forms [7]. CT and MRI findings are similar to ultrasound findings. CT is the imaging study of choice in urgent situations. MRI is used as a complement to CT to further the diagnostic investigation of an adnexal mass [10]. In our case, the typical imaging feature of intracystic fat components was not observed. In our patient, the twisted mature cystic teratoma was depicted in ultrasound as a solid heterogeneous lesion without cystic components and without the typical imaging feature of fat components. It is currently considered that atypical ultrasound features, such as the lack of intracystic fat components or the detection of fat components in atypical locations of the cyst (only within the cyst wall) should be taken seriously in the diagnostic approach to mature cystic ovarian teratomas. The above is a requirement before mature cystic teratomas can be excluded from the differential diagnosis of other ovarian masses [11].

Furthermore, Doppler imaging of the pelvis is an important complementary ultrasound technique in the preoperative diagnostic approach of mature cystic teratoma after torsion of the ovarian vascular pedicle. The unilateral significant increase in the ovarian volume due to localized edema caused by venous stasis, the coexisting mass within the affected ovary, the presence of various concentrations of free fluid in the pouch of Douglas, and the lack of arterial or venous flow in the affected adnexa significantly increase the probability of adnexal torsion. However, it is useful to underline that the presence of flow on color Doppler ultrasonography cannot exclude adnexal torsion because it is possible to have torsion of the vascular pedicle with a viable ovary [12].

Preoperative differential diagnosis between pelvic inflammatory disease and adnexal torsion with mature cystic ovarian teratoma in our patient was challenging. Rare cases of malignant transformation of mature cystic ovarian teratoma have been described in the literature, in which abdominal pain and vomiting were incorrectly attributed to adnexal torsion [13,14]. In addition, in rare isolated cases, mature cystic teratoma may coexist with ovarian lymphangioma or plasmablastic lymphoma with all the difficulties related to the correct diagnosis [15,16]. Cases of mature cystic ovarian teratoma misdiagnosed as bladder diverticulum have also been described in the literature [17]. Furthermore, a differential diagnostic challenge may be caused by the torsion of mature cystic ovarian teratoma during the early postpartum period [18].

The treatment is surgical. Torsion of mature cystic ovarian teratoma usually concerns patients of reproductive age who wish to preserve fertility and more frequently occurs in premenopausal women, but it can also happen to women of postmenopausal age. This makes the minimally invasive approach the optimal treatment option for these patients [19]. Early diagnosis and treatment are vital for good clinical outcomes and preservation of the ovary and/or fallopian tube [20]. In our patient, atypical clinical and imaging findings significantly delayed the correct diagnosis. The misdiagnosis resulted in adnexectomy and loss of the ipsilateral fallopian tube and ovary.

Cystectomy is considered to be the treatment of choice in young women who wish to preserve ovarian function [21]. Confirmation of the diagnosis of dermoid cyst should be determined postoperatively by histological examination of the operative specimen. On macroscopic examination, dermoid cysts are usually polycystic and contain sebaceous fluid and tissue of two or more layers of germ cells, such as hair (ectoderm) or teeth, bone, muscle, fat (mesoderm) or mucosa (endoderm) [22]. In our patient, the presence of hair follicles and fatty tissue without evidence of malignancy has set with precision the diagnosis of mature cystic ovarian teratoma.

Adnexectomy (unilateral or bilateral) and hysterectomy, depending on the patient’s age, may be considered necessary in cases of complete adnexal torsion, making the ovary and fallopian tube non-viable [23]. The laparoscopic approach is currently widely accepted, although cyst rupture and spillage rates into the peritoneal cavity are quite high (15% to 100%) compared to laparotomy (4% to 13%) [21]. A recent study demonstrates that the laparoscopic surgical approach is safe even in those cases characterized by the rupture of the dermoid cyst and spillage of its contents into the peritoneal cavity [24].

## Conclusions

Torsion of mature cystic ovarian teratoma can be misdiagnosed as a pelvic inflammatory disease. Adnexal torsion, particularly when accompanied by atypical clinical symptoms and imaging findings, can create a serious differential diagnostic challenge and significantly delay correct diagnosis. Early diagnosis and treatment should be a primary concern of modern obstetricians and gynaecologists, especially when it comes to young patients who wish to preserve fertility and achieve future pregnancy.
